# Supplementary material for: Estimating the number and percentage of children who experience parental incarceration in Canada using whole population administrative and vital statistics data
Source: PLoS One. 2026 Apr 8;21(4):e0344941. doi: 10.1371/journal.pone.0344941 (PMC13061208; doi:10.1371/journal.pone.0344941)
Supplement: S1 File — Estimation of the number of children who experienced parental incarceration per day and per year in Canada. (DOCX) [file pone.0344941.s001.docx]

**S1 File. Appendix A.** Estimation of the number of children who experienced parental incarceration per day and per year in Canada

**Table 3. Estimation of children who experienced parental incarceration in Canada per day**

| **Source** | **Indicator** | **2016** | **2017** |
| --- | --- | --- | --- |
| **Study data** | A. Total children who experienced parental incarceration/day as identified in the study | 10,841 | 12,649 |
|  | B. Total adults incarcerated/day included in the study | 16,740 | 17,750 |
| **Statistics Canada^a 40,41^** | C. Total adults incarcerated in Canada/day | 39,872.8 | 38,809.0 |
| **Derived** | D. Total children who experienced parental incarceration/day in Canada: Applying the ratio of children who experienced parental incarceration/day to adults incarcerated/day from the study to the total adults incarcerated in Canada/day (A/B x C) | 25,822.0 | 27,656.1 |
| **Statistics Canada^31^** | E. Total children in Canada | 4,693,143 | 4,718,582 |
| **Derived** | F. Percentage of children in Canada who experience parental incarceration (Dx100/E) | 0.55% | 0.59% |

^a^Statistics Canada reports these data by fiscal vs. calendar year. We used 2016/17 data for 2016 and 2017/18 data for 2017 estimates. For 2016/17, there was an average of 25,447.5 people incarcerated in provincial correctional facilities and 14,425.3 in federal correctional facilities per day, and for 2017/18, there was an average of 24,680.5 people incarcerated in provincial/territorial correctional facilities and 14,128.5 incarcerated in federal correctional facilities per day.

**Table 4. Estimation of children who experienced parental incarceration in Canada per year**

| **Source** | **Indicator** | **2016** | **2017** |
| --- | --- | --- | --- |
| **Study data** | A. Total adults incarcerated/day in PT facilities included in the study | 16,740 | 17,750 |
| **Statistics Canada^40,41^** | B. Total adults incarcerated/day in PT facilities in Canada | 25,447.5 | 24,680.5 |
| **Derived** | C. Proportion of adults incarcerated/day in PT facilities identified in the study (A/B) | 0.66 | 0.72 |
| **Study data** | D. Total adults incarcerated in PT facilities/year in the study | 79,770 | 81,910 |
| **Derived** | E. Total adults incarcerated in PT facilities/year in Canada: Using the proportion of incarcerated adults per day identified in the study to adjust the total adults incarcerated/year in the study to reflect the Canadian total (D/C) | 121,263.3 | 113,891.8 |
| **Statistics Canada^40,41^** | F. Total adults incarcerated in federal facilities in Canada/year (estimated^a^) | 14,425.30 | 14,128.50 |
| **Derived** | G. Total adults incarcerated in Canada/year (E+F) | 135,688.57 | 128,020.32 |
| **Study** | H. Total children who experienced parental incarceration/year in the study | 54,580 | 60,590 |
| **Derived** | I. Total children who experienced parental incarceration/year in Canada: Applying the ratio of children who experienced parental incarceration/year to adults incarcerated/year from the study to the total adults incarcerated in Canada/year (H/D x G) | 92,840.4 | 94,698.5 |
| **Statistics Canada^31^** | J. Total children in Canada | 4,693,143 | 4,718,582 |
| **Derived** | K. Percentage of children in Canada who experience parental incarceration (I x 100/J) | 1.98% | 2.01% |
| **Statistics Canada^31^** | L. Total population in Canada | 36,110,803 | 36,545,075 |
| **Derived** | M. Rate of children who experience parental incarceration in Canada per year per 100,000 population (I x 100,000/L) | 257.1 | 259.1 |

^a^Since the length of incarceration for people in federal correctional facilities is 2 or more years, we used the number of people incarcerated in federal facilities per day as a conservative estimate of the number of people incarcerated in federal facilities per year.
