# Supplementary material for: Estimating the number and percentage of children who experience parental incarceration in Canada using whole population administrative and vital statistics data
Source: PLoS One. 2026 Apr 8;21(4):e0344941. doi: 10.1371/journal.pone.0344941 (PMC13061208; doi:10.1371/journal.pone.0344941)
Supplement: S2 File — (PDF) [file pone.0344941.s002.pdf]

Hamilton Integrated Research Ethics Board

Date: May-31-2023

Local Principal Investigator: Dr. Fiona Kouyoumdjian

Participating HiREB Centre(s): McMaster University

Project ID: 16201

Project Title: Estimating the number of children who experience parental incarceration and describing their health status: The CHIRP (Children with IncarceRated Parents) Study

Review Type: Delegated

Date of Final Approval: May-28-2023

Ethics Expiry Date: May-28-2024

The Hamilton Integrated Research Ethics Board (HiREB) has reviewed and approved the abovementioned study.

The following documents have been approved:

Document Name	Document Date	Document Version
Protocol for HIREB March 6 2023	Mar-06-2023	1
Data collection form for HIREB March 6 2023	Mar-06-2023	1
Letter for HIREB May 26 2023	May-26-2023	1

The following documents have been acknowledged:

Document Name	Document Date	Document Version
Kouyoumdjian LoS - May 2023	May-26-2023	1

While HiREB has reviewed and approved this application, the research must be conducted in accordance with applicable regulations and institutional and/or public health requirements.

We are pleased to issue **final approval** for the above-named study until the expiry date noted above. Continuation beyond that date will require further review and renewal of REB approval. Any changes or amendments to the protocol or study documents must be approved by the Hamilton Integrated Research Ethics Board.

Conditions: You are only permitted to record the data that you have included in your approved Data Collection Form.

If you require a listing of chart identifiers for this project, please contact the Decision Support Services department who will be able to assist you with this data extraction. There will be a charge for data extraction so please discuss your requirements to enable the assigned analyst to generate an estimate of workload. Decision Support Services will require your written consent to pay prior to commencing with the data extraction. Please allow 4-6 weeks for the completion of the request after your consent to pay is received. If you have any questions regarding the billing and consent process, you should discuss this as well.

Once you have the chart identifiers and you require access to the patient's health records through the Health Records Department at Hamilton Health Sciences, please submit the listing as follows:

MUMC, Juravinski Hospital, Juravinski Cancer Centre, Hamilton General, Chedoke and St. Peters, contact:
Mike Taylor, Manager, Health Records, ext 76767.

If you require any type of computer assistance, including passwords, please contact the Hamilton Health Sciences ICT department at ext 43000.

REB members involved in the research project do not participate in the review, discussion or decision.

The Hamilton Integrated Research Ethics Board (HiREB) provides ethical review and ongoing ethical oversight on behalf of Hamilton Health Sciences, St. Joseph's Healthcare Hamilton, Research St. Joseph's-Hamilton, the Faculty of Health Sciences at McMaster University and Niagara Health. HiREB operates in compliance with and is constituted in accordance with the requirements of: The Tri-Council Policy Statement on Ethical Conduct of Research Involving Humans (TCPS 2); The International Conference on Harmonisation of Good Clinical Practices Guideline (ICH GCP); Part C Division 5 of the Food and Drug Regulations, Part 4 of the Natural

Health Products Regulations; Part 3 of the Medical Devices Regulations and the provisions of the Ontario Personal Health Information Protection Act 2004 and its applicable Regulations. For studies conducted at St. Joseph's Healthcare Hamilton, HiREB complies with the Health Ethics Guide of the Catholic Alliance of Canada. HiREB is qualified through the Clinical Trials Ontario (CTO) REB Qualification Program and is registered with the U.S. Department of Health and Human Services (DHHS) Office for Human Research Protection (OHRP).

Sincerely,

Frederick A Spencer

Dr. Frederick A. Spencer, MD
Chair, Hamilton Integrated Research Ethics Board

Hamilton Integrated Research Ethics Board (HiREB)
237 Barton Street, Suite C1-205   Hamilton, ON   L8L 2X2
Telephone: 905-521-2100, Ext. 42013
