# Supplementary material for: Estimating the number and percentage of children who experience parental incarceration in Canada using whole population administrative and vital statistics data
Source: PLoS One. 2026 Apr 8;21(4):e0344941. doi: 10.1371/journal.pone.0344941 (PMC13061208; doi:10.1371/journal.pone.0344941)
Supplement: Attachment — Submitted filename: Response_to_reviewers_auresp_2.docx [file pone.0344941.s005.docx]

**Response to editors’ comments**

*I am so enthusiastic about this paper and am eager to see it go to "print." I appreciate your detailed responses to the reviewers comments. I have identified one instance where I believe that the text that is in that response would additionally strengthen the manuscript, and one potential typographical error. Once these can be addressed, I look forward to accepting the manuscript.*

We appreciate these positive comments.

*1. In the response to reviewers, you mention using the death data to calculate time at risk of parental incarceration and the percent of childhood with a parent incarcerated for children. Please include this detail in the methods section around line 205 when the descriptive analysis is mentioned.*

We have added this content as requested.

*2. Should line 270 read "<18 years old" instead of "≥18 years old"? I'm not certain, but I can't make sense of it as written.*

Thank you for catching this error, and we have corrected the text to say <18 years old.
